# *Salix alba* attenuated oxidative stress in the heart and kidney of hypercholesterolemic rabbits

**Published:** 2018

**Authors:** Narges Amel Zabihi, Maryam Mahmoudabady, Mohammad Soukhtanloo, Parichehr Hayatdavoudi, Farimah Beheshti, Saeed Niazmand

**Affiliations:** 1 *Department of Physiology, Faculty of Medicine, Mashhad University of Medical Sciences* *, * *Mashhad, Iran *; 2 *Neurogenic Inflammation Research Center, Mashhad University of Medical Sciences, Mashhad, Iran*; 3 *Department of Clinical Biochemistry, School of Medicine, Mashhad University of Medical Sciences, Mashhad, Iran*; 4 *Cardiovascular Research Center, Mashhad University of Medical Sciences, Mashhad, Iran*

**Keywords:** Hypercholesterolemia, Salix alba, Oxidative stress, Heart, Kidney, Rabbit

## Abstract

**Objective:**

Oxidative stress plays a critical role in the development of hypercholesterolemia-induced complications. This study evaluated the effects of aspirin and *Salix alba* hydroethanolic extract on oxidative stress in the heart and kidney of hypercholesterolemic rabbits.

**Materials and Methods:**

The antioxidant activity, as well as total phenolic and salicin content of *S. alba *(Sa) extract were assessed by DPPH radical scavenging activity, Folin-Ciocalteu and HPLC methods, respectively. Animals were divided into two groups of control (fed with normal chow), and HD (fed with high cholesterol diet for 6 weeks). Then, hypercholesterolemic animals allocated to the following treatment groups: CHO (received HD), Sa extract (HD plus extract 60 and 120 mg/kg), and aspirin (HD plus aspirin 120 mg/kg) and received the treatments on a daily basis for 6 weeks. MDA, GSH, and nitrite concentrations as well as the activities of SOD and CAT were evaluated in cardiac and kidney tissues.

**Results:**

The scavenging activity, total phenolic content and salicin were 19.1 µg/ml (IC50), 153.75 ± 3.6 mg of gallic acid/g, and 18.03 µg/mg, respectively. In comparison to CHO group, MDA levels were diminished in Sa and ASA groups but GSH levels were improved. NO metabolites increased in the heart of Sa 120 mg/kg group and in the kidney of all Sa and ASA treated groups. SOD activity increased only in the heart of Sa groups and in the kidney of Sa and ASA groups. CAT activity increased in the heart and kidney tissues of all Sa and ASA treated groups.

**Conclusion:**

The results showed *S. alba *extract improved redox homeostasis in heart and kidney tissues of hypercholesterolemic rabbits. The extract antioxidant property may be related to its phenolic content.

## Introduction

Clinical studies have indicated that hypercholesterolemia affects a significant number of adults in developed countries (Kuklina et al., 2009 ▶; Felix-Redondo et al., 2013 ▶). It is well recognized that this metabolic disturbance is a strong risk factor for cardiovascular diseases (CVD) (Nelson 2013 ▶) and is associated with renal damage (He et al., 2015 ▶). Clinical and experimental evidence established that hypercholesterolemia is associated with inflammation and oxidative stress. For instance, raised F2-isoprostanes and oxygen free radicals has been detected in the urine of patients with high serum cholesterol (5) or in the arteries of hypercholesterolemic animals (6).

According to experimental evidence, oxidative stress and inflammation are key phenomena in various clinical conditions. Therefore, enhancing antioxidant supply in hypercholesterolemia subjects may help to prevent the consequences. Aspirin (acetylsalicylic acid (ASA)) is a cardiovascular protective medication and it is well-known for reducing the risk of secondary and primary cardiovascular events. Aspirin partially exerts its protective effects via inhibiting cyclooxygenase-1 (COX-1), modifying COX-2 activity, and stimulating the production of endogenous anti-inflammatory mediators including lipoxins, which diminish the inflammatory response and reduce the levels of inflammatory biomarkers, including C-reactive protein (CRP). Moreover, aspirin has free radical-scavenging properties and could protect endothelial cells from oxidative damage (Podhaisky et al., 1997 ▶). In addition to the anti-inflammatory and antioxidant properties, lipid lowering effect of high dose aspirin (120 mg/kg) has been reported (Benigni et al., 2010 ▶; Pacurari et al., 2014 ▶).

White willow (*Salix alba *L.), belonging to the genus *Salix* and Salicaceae family, has a very long history of medicinal usage, dating back to nearly 6000 years (Sumner 2000 ▶). The bark and leaf of willow species contain the salicin prodrug which was identified in 1829 by the French pharmacist H. Leroux (Csonka et al., 2015 ▶). More than 80% of salicin content is absorbed and then metabolized into different salicylate derivatives (Steinegger and Hövel 1972 ▶). Besides the salicin-related constituents, other active components of willow leaves and bark such as polyphenols and flavonoids play important roles in therapeutic effects of willow (Nahrstedt et al., 2007 ▶; Shara and Stohs 2015 ▶). It has been indicated that a polyphenolic compound isolated from willow bark, 2, 3-trans procyanidin, induces relaxation in porcine coronary arteries through activation of PI3/Akt kinase signaling pathway and eNOS phosphorylation (Kaufeld et al., 2013 ▶). Also, various *in vitro* studies have shown antioxidant activity of willow extract (Bonaterra et al., 2010 ▶; Enayat and Banerjee 2009 ▶; Jukic et al., 2012 ▶). It was reported that in human vascular endothelial cells, willow bark extract could induce antioxidant enzymes and prevent oxidative stress through activation of nuclear factor erythroid 2-related factor 2 (Nrf2) (Ishikado et al., 2013 ▶). In diabetic rats, a fortified extract containing *S. alba* could reduce lipid peroxidation and inflammatory cytokines levels (Bucolo et al., 2013 ▶). The antioxidant effect of standardized willow bark extract was demonstrated in serum of rats with adjuvant-induced arthritis (Khayyal et al., 2005 ▶). In addition to antioxidative and anti-inflammatory properties, willow bark is used in weight loss supplements (Shara and Stohs 2015 ▶). Moreover, in normolipidemic rabbits, aromatic water of *Salix* *aegyptiaca* decreased level of total cholesterol and this effect was comparable to that of simvastatin (Karimi et al., 2015 ▶)

The aim of the present study was to evaluate the antioxidative potency of *S. alba* (white willow) in comparison to ASA in heart and kidney tissues of diet -induced hypercholesterolemic rabbits.

## Materials and Methods

**Plant collection and extract preparation**

Leaves, first-year twigs and stems of *S. alba* were collected in March from Bojnourd, North Khorasan, Iran, identified by botanists, and kept in the herbarium of Ferdowsi University of Mashhad (voucher No. 45364). Immediately after collection, the leaves and young stems were washed and dried at ambient temperature. Dried leaves and stems grounded well and soaked in 2 L of ethanol (70%) for 72 hr at 4 °C (Pantelidis et al., 2007 ▶). Then, the mixture was filtered through a paper filter and subjected to rotary evaporation under vacuum at 40 °C until the solvent was evaporated. Next, water was removed by vacuum lyophilization and then the lyophilized extract was kept at -80 °C until use. 

**Phytochemical studies**

*Determination of salicin content in samples*

Salicin content of extract was detected by a gradient reverse-phase high performance liquid chromatography (RP-HPLC) with UV detection. The chromatographic system consisted of a Nucleosil column (250 × 4.6 mm; C18 5 μm). The mobile phase consisted of water/methanol with a gradient program at a flow rate of 1 ml/min. Detection was done at 268 nm and an injection volume of 20 μl was used.

Determination of total phenolic content

Total phenolic contents (TPC) were estimated using the method of Singleton and Rossi (Singleton and Rossi 1965 ▶). In brief, 200 µL of the extract (1mg/ml) was mixed with 10 ml of 1:10 folin-ciocalteu reagent and after a period of 5 min, 7 ml of Na_2_CO_3_ solution (0.115 mg/ml) was added. The samples were vortexed and incubated for 2 hr and absorbance was read at 765 nm. Then, a standard calibration curve of gallic acid was prepared (0.000469X + 0.02689, r = 0.9976). Results were expressed as gallic acid equivalents (GAE) (mg gallic acid/g dry extract). Data was recorded in triplicate.

*DPPH (1, 1-diphenyl-2-picryl-hydrazyl) Scavenging Assay*

*In vitro* free-radical scavenging activity of the Sa sample was measured according to Brand-Williams method (Brand-Williams et al., 1995 ▶), with some modifications. Briefly, Sa (1 ml) (1–180 μg/ml) was added to 2 ml of a DPPH solution in methanol (0.004%). The mixture was shaken well and left for 30 min in the dark at room temperature. The absorbance (*A*_sample_) of the resulting solution was measured at 517 nm and the percentage of antioxidant activity (AA%) was calculated using the following formula: AA% = 100 − {[(*A*_sample_ − *A*_blank_) × 100]/*A*_control_}. A mixture of methanol (2 ml) and Sa (1 ml) was used as blank (*A*_blank_), while a solution of DPPH (2 ml) and methanol (1 ml) was taken as control (*A*_control_). Ascorbic acid (ASA) and BTH (Butylated hydroxy toluene) were used as standards at the same concentrations as Sa. Free radical-scavenging activity was expressed as the quantity of antioxidants necessary to decrease the initial DPPH absorbance by 50% (IC_50_). The IC_50_ value was calculated by Graphpad Prism software. 

**Chemicals and drug**

All chemicals were of analytical grade (Merck). Salicin (Sigma), GSH (Sigma), folin-ciocalteu reagent (Sigma), DDPH (Sigma), Cholesterol (Daejung, South Korea) and aspirin were gifts from Temad Co. 

**Animals**

Twenty-five male New Zealand rabbits (1.8 - 2 kg) were purchased from Pasteur Institute, Iran. They were individually housed in cages under standard conditions with 12hr-12hr light-dark cycle at 22 ± 2 °C with free access to food and water. All procedures were approved by the Ethical Committee of the Mashhad University of Medical Sciences, Mashhad, Iran (approval No. 900910). 

**Experiment design**

After 2 weeks of acclimation to a commercial diet, rabbits were randomly divided into 2 groups: 1) control group fed with standard chow diet (n=5), 2) hypercholesterolemic group (CHO) fed with a high- cholesterol diet (0.5% w/w cholesterol) for 6 weeks to provoke the atherosclerosis process. Induction of hypercholesterolemia was confirmed by measuring cholesterol levels in blood samples taken from the lateral saphenous veins. Hypercholesterolemic rabbits were randomly divided into the following groups and daily received a single dose of the following supplementation by gavage for 6 weeks while control group received normal saline: 

1. CHOL group: hypercholesterolemic diet plus normal saline, 2. Sa 60 group: hypercholesterolemic diet plus *S. alba* extract 60 mg/kg, 3. Sa 120 group: hypercholesterolemic diet plus *S. alba* extract 120 mg/kg, 4. ASA group: hypercholesterolemic diet plus aspirin 120 mg/kg. 

**Preparation and analysis of the samples**

At the end of the treatment period, rabbits were sacrificed under deep anesthesia (thiopental 60 mg/kg. i.v). Then, the heart and kidney were excised rapidly and rinsed in cold normal saline. The heart and kidney tissues were homogenized in 10 % (w/v) homogenizing buffer (100 mM KH_2_PO_4_, K_2_HPO_4_, pH 7.4). The homogenate tissues were centrifuged at 860 g for 15 min at 4 °C, and the resultant supernatants were used for different assays.

**Biochemical assays**

Catalase (CAT) and Super Oxide Dismutase (SOD) activity were measured using a kit (Cayman Co, USA), and nitrite levels (stable NO metabolite) were measured using a colorimetric kit (Promega Co, USA). 

*Determination of GSH*

Reduced glutathione (GSH) was measured using Ellman's reagent based on the method of Moron (Moron et al., 1979 ▶). Briefly, 1.8 ml of 0.2 M Na_2_HPO_4_ was mixed with 40 µl of 10 mM DTNB and 160 µl of the supernatant. It was left for 2 min and the absorbance was read at 412 nm. A solution of 0.001 M of GSH was used as standard.

*Determination of MDA*

MDA, used as an index of lipid peroxidation, reacts with thiobarbituric acid (TBA) as a TBA reactive substance (TBARS), and produces a redish complex which has a peak absorbance at 535 nm. Briefly, 1 mL of supernatant was added to 2 mL of a solution consisting of TBA, trichloroacetic acid (TCA), and hydrochloric acid (HCl). The mixture was stirred and heated on a boiling water bath for 45 min. After cooling, 4 ml of n-butanol was added, shaken, and centrifuged to separate the butanol layer. The absorbance was read at 535 nm (Ohkawa et al., 1979 ▶).

**Statistical analysis**

Results are expressed as mean ± SEM. Statistical analyses were done using one-way ANOVA followed by the LSD test. Statistical significance was defined as p<0.05.

## Results

**Extract analysis**

*Total phenolics and salicin contents*

**Figure 1:**
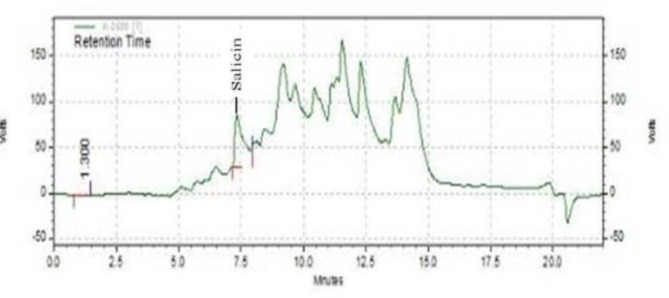
RP-HPLC chromatogram of *salix alba* extract. Chromolith Performance Nucleosi (250 × 4.6 mm; C18 5 μm) column – mobile phase: water/methanol, v = 1.2 mL/min, UV detection at λ =265 nm

Total phenolic content was found to be approximately 153.75 ± 3.6 mg GAE/g of dry extract and salicin was about 18.03 µg/mg dry weight. (Figure. 1)

*DPPH radical scavenging activity*

**Figure 2:**
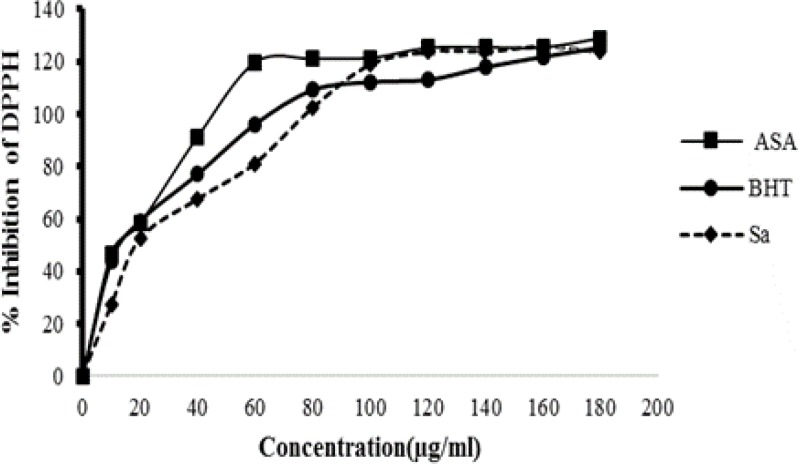
2, 2-Diphenyl-1-picrylhydrazyl (DPPH) radical scavenging activity of *Salix alba* hydroethanolic extract, as well as that of ascorbic acid and Butylated hydroxy toluene (BHT) as the standards. (n=3

The scavenging activities of Sa extract was determined using free radicals of 1, 1-diphenyl 1-2-picryl-hydrazyl (DPPH). The highest scavenging effect was observed for Sa extract with an IC_50_ of 19.1 μg/ml. However, Sa scavenging activity was lower than that of ascorbic and BHT, which were used as standards (12.41µg/ml and 13.53 μg/ml, respectively) (Figure. 2).

**SOD and CAT activities**

**Figure 3:**
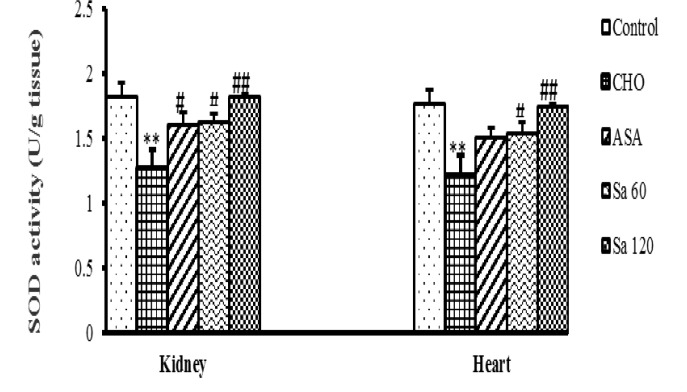
Superoxide dismutase (SOD) activity in kidney and heart tissues. CHO: hypercholesteremic group, ASA: the group treated with aspirin 120mg/kg, Sa 60: the group treated with *Salix alba* extract 60 mg/kg, and Sa 120: the group treated with *Salix alba* extract 120 mg/kg. Values are means ± SEM (n = 5).

SOD activity in the heart and kidney tissues of CHO group was lower than that of control group (p<0.005). Treatment of the animals with the extract significantly increased SOD activity in the heart, as compared to CHO group (p<0.001-0.05). SOD activity in kidney tissues of all treated groups increased significantly (p<0.05-p<0.001), (Figure. 3).

**Figure 4:**
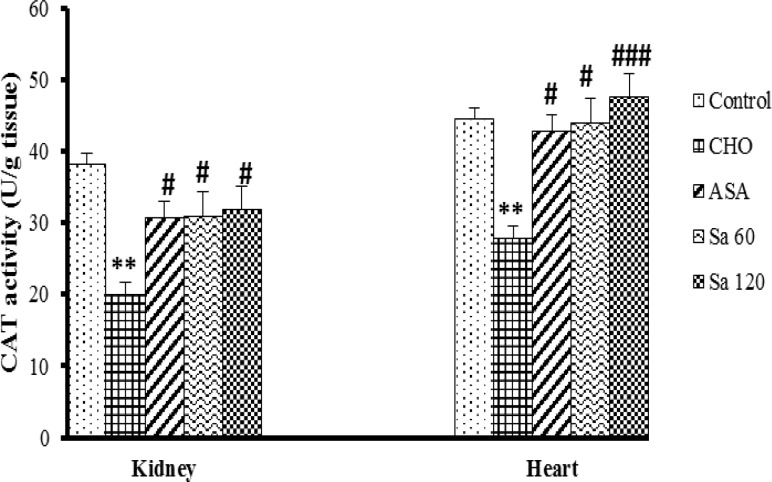
Catalase (CAT) activity in kidney and heart tissues. CHO: hypercholesteremic group, ASA: the group treated with aspirin 120 mg/kg, Sa 60: the group treated with *Salix alba* extract 60 mg/kg, and Sa 120: the group treated with *Salix alba* extract 120 mg/kg. Values are means ± SEM (n = 5). ** p<0.01 compared to control group. **# **p<0.05 and ### p<0.001 as compared to hypercholesterolemic group. Statistical analyses were done using one-way ANOVA followed by the LSD’s test

In addition, CAT enzyme activity in the heart of CHO group was lower than that of control group (p<0.05). Treatment of the animals with Sa 60, Sa 120 and ASA increased CAT activity in the heart tissues in comparison to CHO (p<0.001- 0.05). In the kidney tissues, activity of CAT enzyme in CHO group was lower than that of control group (p<0.001). However, treatment of the animals with Sa 60, Sa 120 and ASA increased CAT activity in the kidney tissues in comparison to CHO group (p<0.05), (Figure. 4).

**GSH**, **nitrite and MDA concentration **

**Figure 5:**
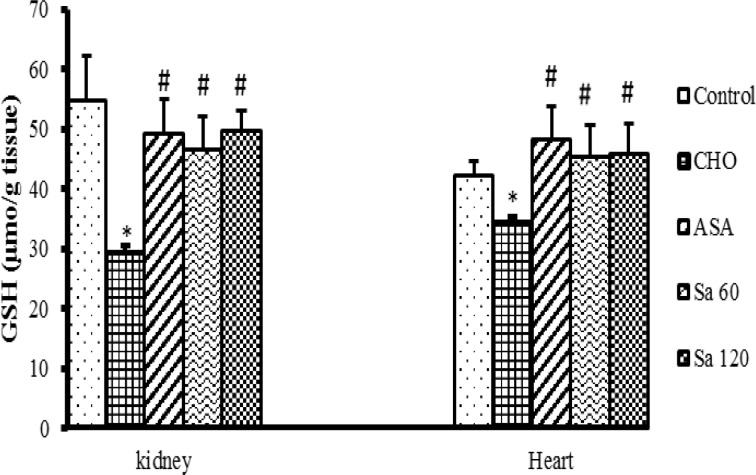
The reduced glutathione (GSH) levels in kidney and heart tissues. CHO: hypercholesteremic proup, ASA: the group treated with ASA (aspirin 120 mg/kg), Sa 60: the group treated with *Salix alba* extract (60 mg/kg), Sa 120: the group treated with *Salix alba* extract (120 mg/kg). Values are means ± SEM (n = 5).

GSH content in the heart and kidney tissues of CHO group was lower than that of control group (p<0.05). Treatment with ASA and or Sa extracts increased the GSH content (p<0.05) (Figure. 5).

* p<0.05 compared to control group and **# **p<0.05 compared to hypercholesterolemic group. Statistical analyses were made using the one-way ANOVA followed by the LSD’s test.

**Figure 6:**
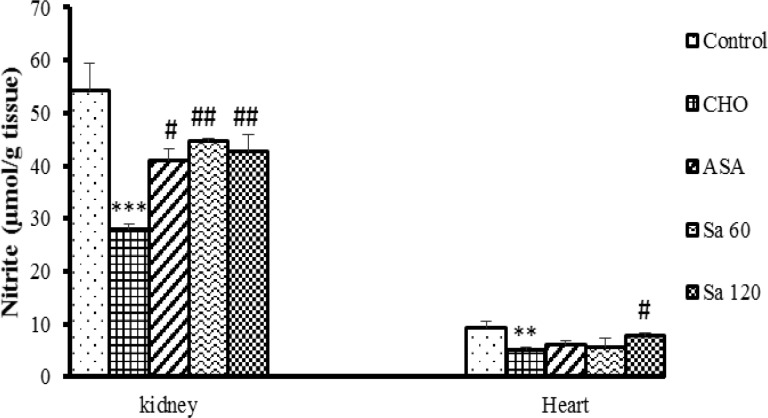
Nitrite levels in kidney and heart tissues. CHO: hypercholesteremic group, ASA: the group treated with aspirin 120 mg/kg, Sa 60: the group treated with *Salix alba* extract 60 mg/kg, and Sa 120: the group treated with *Salix alba* extract 120 mg/kg. Values are means ± SEM (n = 5). ** p<0.01 and *** p<0.001 as compared to control group. **# **p<0.05 and ## p<0.01, as compared to hypercholesterolemic group. Statistical analyses were done using one-way ANOVA followed by the LSD’s test

Nitrite (NO metabolite) concentration in the heart tissues of CHO group was lower than that of control group (p<0.01). In Sa 120 group, NO metabolites increased in the heart tissues in comparison to CHO group (p<0.05).Nitrite concentration in renal tissues of CHO group was lower than that of control group (p<0.001). Nitrite in the kidney tissues of ASA and extract-treated groups was higher than that of CHO group (p<0.01-p<0.001) (Figure. 6).

**Figure 7:**
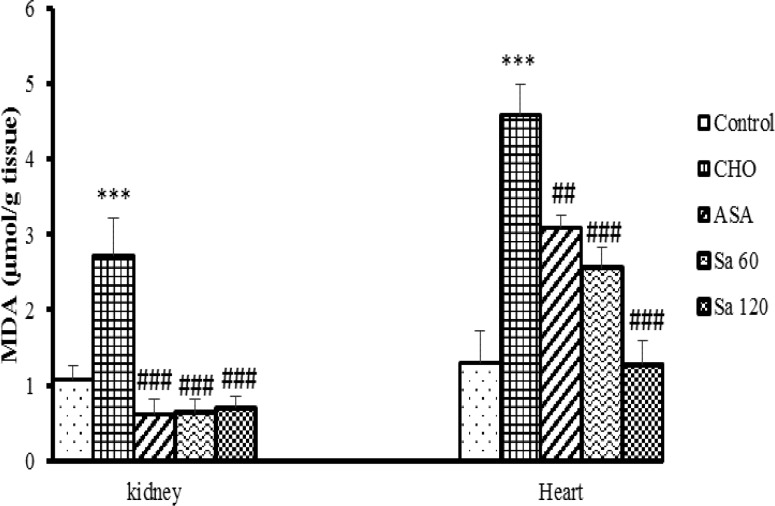
Malondialdehyde (MDA) levels in kidney and heart tissues CHO: hypercholesteremic proup, ASA: the group treated with aspirin 120 mg/kg, Sa 60: the group treated with *Salix alba* extract 60 mg/kg, and Sa 120: the group treated with *Salix alba* extract 120 mg/kg. Values are expressed as means ± SEM (n = 5).

 MDA concentration in the heart and kidney tissues of CHO group was higher than that of control group (p<0.001). Treatment with extract and ASA decreased MDA concentration in the heart and kidney tissues in comparison to CHO group (p<0.01-0.001), (Figure. 7).

## Discussion

The aim of this experimental study was to determine the protective effect of *S. alba* extract and aspirin against hypercholesterolemia-induced oxidative stress in rabbits heart and kidney tissues. Oxidative stress is an initial crisis in hypercholesterolemia (Bonaterra et al., 2010 ▶; Csonka et al., 2015 ▶), and the cornerstone of various diseases including cardiovascular events (Bonaterra et al., 2010 ▶; Sulaiman et al., 2013 ▶), and kidney injuries (Moron et al., 1979 ▶). Oxidative imbalance is established following depletion of enzymatic and non-enzymatic antioxidants, increase in lipid peroxidation and decrease in NO levels. Lipid peroxidation (LPO) is triggered by a hydroxyl radical, leading to a free radical chain reaction and ultimately membrane breakage. MDA is used as a marker of LPO. Intracellular thiols such as glutathione (GSH) play an important role in maintaining the redox environment inside the cell. When cells are exposed to oxidative stress, thiol groups are the first antioxidants that are consumed (Jones et al., 2000 ▶). As obtained results indicated, diet-induced hypercholesterolemia  resulted in statistically significant changes in redux state of the heart and kidney. Consequently, MDA levels, as an indicator of oxidative damage, increased while the GSH, SOD and CAT levels, as endogenous antioxidants, diminished in the heart and kidney of hypercholesterolaemic rabbits. Moreover nitrite concentrations in hypercholesterolaemic rabbits significantly decreased. *S.* *alba* extract and ASA supplementations effectively improved oxidative stress. The activities of SOD and CAT enhanced and the GSH and NO levels increased, while MDA levels reduced in the hypercholesterolaemic rabbits’ heart and kidney of* S.* *alba* extract and ASA treated groups. 

Cardiovascular protective effects of ASA have been previously established (Brand-Williams et al., 1995 ▶). Therapeutic effects of ASA are not limited to inhibition of prostaglandin synthesis. Various studies have demonstrated that this drug has the ability to inhibit activation of nuclear factor-kappa B, a ubiquitous mediator of inflammatory response (Bonaterra et al., 2010 ▶). ASA is able to improve endothelial dysfunction (Ohkawa et al., 1979 ▶; Singleton and Rossi 1965 ▶) and prevent oxidation of LDL (Oliveira et al., 2008 ▶). ASA treatment significantly restored reduced levels of glutathione and ameliorated lipid peroxidation in hypercholesterolaemic rats (Steinegger and Hövel 1972 ▶). In cardiomyopathic hamsters, long-term treatment with ASA significantly prevented oxidative stress and cardiac hypertrophy (Wu et al., 2012 ▶). ASA extended the life span of *Caenorhabditis elegans* by attenuating endogenous reactive oxygen species and up-regulating superoxide dismutase, catalase, and glutathione S-transferase genes (Ayyadevara et al., 2013 ▶). Also, in middle-aged men, administration of enteric-coated aspirin improved blood antioxidative potency (Kumar and Pandey 2013 ▶). Antioxidant properties of ASA might be due to its free-radical neutralizing ability (Khayyal et al., 2005 ▶). 

In our study, Sa extract at the dose of 120 mg/kg was more efficient than ASA that was in accordance with previous studies (Khayyal et al., 2005 ▶). Our results indicated that only treatment with Sa 120 mg/kg could notably improve NO levels in heart tissues and enhance SOD activity in kidney tissues. This effect of Sa extract could be due to its polyphenolic content and antioxidant activity. Also, Sa extract revealed marked free-radical scavenging activity which was concentration-dependent. Measurement of DPPH radicals scavenging activity is considered as a valid accurate method and is extensively used to determine free-radical scavenging activity of different compounds (Kedare and Singh 2011 ▶; Sulaiman et al., 2011 ▶). A pervious experiment revealed that *S.* *alba* ethanolic extract has antimicrobial activities and it is cytotoxic against human leukemia cell line. The antimicrobial and cytotoxic activities of the extract were positively associated with its antioxidant potentials (Sulaiman et al., 2013 ▶). 

Traditionally, salicin has been considered as the major active constituent of willow bark and little attention has been given to the role of its polyphenols and flavonoids. However, Ishikado et al. study indicated that a salicin -free willow bark extract fraction prompted antioxidant enzymes (Ishikado et al., 2013 ▶), and reduced oxidative stress through activation of nuclear factor erythroid-2 related factor-2 in human umbilical vein endothelial cells and the nematode *C. elegans* (Ishikado et al., 2013 ▶). Antioxidant effects of willow extract have been studied in various experiments. In addition to salicin, willow bark contains various flavonoids and polyphenols that synergistically contribute to the beneficial effects of Sa which may be more marked than ASA (Shara and Stohs 2015 ▶; Sulaiman et al., 2011 ▶). It is well-recognized that polyphenolic compounds possess several pharmacological activities, including anti-oxidant (Kumar and Pandey 2013 ▶), anti-inflammatory and hypolipidemic properties (Dudzińska et al., 2015 ▶). Willow bark also contains several known active compounds such as catechin and amelopsin which have antioxidant and free radical scavenging activities (Abascal et al., 2005 ▶; Orians 1995 ▶). In rats with acute and chronic inflammation, a standardized willow bark extract, which had lower "salicin" content than an equivalent dose of ASA, increased GSH levels and reduced lipid peroxidation even more potent than either ASA or celecoxib (Khayyal et al., 2005 ▶). Moreover, willow extract, compared to aspirin, has minimal adverse effects which may be due to its antioxidants compounds (Pantelidis et al., 2007 ▶), and it is not harmful to the gastrointestinal mucosa (Jones et al., 2000 ▶) probably due to its different effect on COX-1 and COX-2 expression (Bonaterra et al., 2010b ▶). 

In conclusion, the present study has evidently confirmed that ASA and Sa extract exhibit antioxidant property and improved oxidative status in heart and kidney tissues of hypercholosterolemic rabbits. Moreover, Sa extract indicated free-radical scavenging activity against DPPH stable radical. These results may provide a rational for using Sa extract for treatment of certain conditions related to oxidative stress. 
